# Platelet Rich Plasma Injection for Soft Tissue Musculoskeletal Pain

**DOI:** 10.5704/MOJ.2107.014

**Published:** 2021-07

**Authors:** KA Irianto, AH Bakri, NA Kloping

**Affiliations:** 1 Department of Orthopaedics and Traumatology, Universitas Airlangga, Surabaya, Indonesia; 2 Department of Orthopaedics, Surabaya Orthopedic and Traumatology Hospital, Surabaya, Indonesia; 3 Faculty of Medicine, Universitas Airlangga, Surabaya, Indonesia

**Keywords:** soft tissues inflammation, platelet-rich plasma, chronic pain

## Abstract

**Introduction:**

Treating soft tissue injuries can be a challenge for physicians as it can be overlooked which can lead to more problems. In recent studies, the use of Platelet Rich Plasma (PRP) has been gaining popularity for soft tissue injuries because of its benefits and minimal side effects. This study aims to evaluate the effect of PRP injection on various musculoskeletal soft tissues inflammation.

**Material and Methods:**

This is a retrospective study of patients with soft tissue injury who underwent PRP therapy between 2015 and 2018 at an orthopaedic and traumatology hospital. The study collected demographic data including the type of soft tissue injury and Visual Analog Scale (VAS) before and after the PRP injection. Those data were statistically analysed to identify the significance of PRP.

**Results:**

Seventy-six patients were included, predominantly female, middle aged (40-69 years old) and class one obesity. Most of the complaints (61.8%) were in the lower extremity region. Pain improvements measured with mean VAS score were observed in both acute (3.06±1.28 to 0.8±0.65) and chronic (4±1.75 to 0.97±0.62) cases. There was a significant decrease of VAS score before and after the PRP injection (p < 0.000).

**Conclusion:**

PRP injection is able to alleviate both acute and chronic pain in soft tissue injuries without additional analgesic.

## Introduction

Soft tissue pain is frequently overlooked and may lead to debilitating conditions. This problem occurs mainly among people with high physical workload such as athletes^1,2^. Soft tissues generally include muscles, fascia, tendon, cartilage, synovium, fibrous capsules, nerves, and ligament, nearly every element beside bones^1^. Surgery is a prevalent option for patients who have persistent symptoms of pain^2^. However, recent studies are trying to focus on anti-inflammatory regiments with greater benefits than surgery. Currently non-surgical approaches include physiotherapy, activity modification, steroid injections, and non-steroidal anti-inflammatory drugs (NSAID)^3^. Even though steroid injections displayed a positive outcome of pain relief, there were reports of harmful effects such as tendon degeneration and muscle atrophy^4^. One of the suggested natural regiments rich in anti-inflammatory agents is platelet-rich plasma (PRP)^2,4,5^.

PRP is an autologous blood product which contains four to five times more platelets than the normal value^2^. The platelets and leucocytes in the PRP have the capability of releasing numerous growth factors, namely platelet-derived growth factor (PDGF), vascular endothelial growth factor (VEGF), epithelial growth factor (EGF), and transforming growth factor beta (TGF-β). This modality has been investigated intensively to foster healing of various musculoskeletal soft tissue diseases, especially soft tissue pain due to excessive or repetitive activities including tendinopathy and injured muscle. Although PRP injection has minor side effects to a certain degree, the advantages of not having cross reactivity and immune reaction or disease transmission risks are much more valuable. PRP can be the lead alternative for soft tissue pain therapy due to its autologous nature^2,3,6^.

Despite positive reports regarding its efficacy, the use of PRP in specific musculoskeletal soft tissues is limited. This study aims to seek the efficacy of PRP injection on the inflammation of various musculoskeletal soft tissues as a stand-alone therapy regiment.

## Materials and Methods

This retrospective study has been approved by the Internal Institutional Review Board of Surabaya Orthopaedic and Traumatology Hospital. Prior to drawing blood, informed consent was obtained from each patient. Patients’ data from between January 2015 and December 2018 were extracted from medical records. The inclusion criteria for the study were adult patients who were diagnosed with musculoskeletal soft tissue inflammation, treated with PRP injection, and had their pain measured with VAS before and after the surgery. Meanwhile, patients with incomplete medical record and who had received additional analgesic after PRP injection were excluded from the study. Demographic data such as subject’s age, sex, body mass index (BMI), duration of pain, confounding disease, and site of lesion were also added. In this study, patients’ BMI were classified based on Asia-Pacific population classification as underweight (<18.5), normal (18.5 – 22.9), overweight (23 – 24.9), obese I (25 – 29.9), and obese II (> 30).

Soft tissue inflammation is defined as non-infectious, non-trauma inflammation involving musculoskeletal structures other than the bone or joint. All soft tissue inflammation cases were diagnosed by orthopaedic surgeons and were grouped into upper and lower extremity based on their anatomical location. There were no limitations of the type of soft tissue inflammation. During the duration of the complaints, samples were categorised into acute pain (< 6 weeks) and chronic pain (> 6 weeks).

The process of collecting PRP was conducted by the hospital laboratory. First, we collected 10 cc of the patient’s peripheral blood. Then the blood was processed and separated in a tube of Z-gel containing ACD (Adenine Citrate Dextrose) anticoagulant using high-speed centrifuge (RCF 2500 G) for 10 minutes. Three separate layers were produced: plasma, buffy coat (platelet), and red blood cells. The thick buffy coat layer, rich in platelet and growth factors, were collected and directly injected within 5 minutes to the subcutaneous tissue of the inflammation site. The bump was slowly massaged to help spread the PRP around the inflammation site. In this study, we made sure to avoid injecting tendon, ligament, as well as the vascular region in the inflammation site.

VAS was measured to define pain before and after the injection. Pain was categorised based on the scale range of 0 (no pain), 1-3 (mild pain), 4-6 (moderate pain), and 7-10 (severe pain). The post-treatment pain score was measured during the patients’ following visit, ranging from 3 weeks to 3 months. Quantitative data were described in terms of mean ± standard deviation. Statistical analysis was performed with SPSS software [version 23.0; SPSS Inc, Chicago, IL, USA]. Paired Samples T-Test was used to determine the significant difference of VAS on pre-post PRP injection. A value of p < 0.05 indicates the result is statistically significant.

## Results

A total of 134 patients were first included in this study. However, 58 samples were excluded as the patients had received additional analgesic after injection. The final total was 76 subjects. The participants were primarily female (55.3%), middle-aged (40 - 69 years), and classified as obese I (42.1%). More than half (56.6%) had confounding disease, and 59.2% were in chronic pain. The baseline characteristics of the subjects are presented in Table I. The distribution of the type of musculoskeletal soft tissue lesions were similar in both acute and chronic cases. Lateral epicondylitis (tennis elbow) is the most frequent case in the upper extremity group, whereas plantar fasciitis is the most prevalent case in the lower extremity group (Fig. I). Other cases in the upper extremity group included common extensor tendon tear, rotator cuff syndrome, subscapularis partial tear, and a particular case of both supraspinatus tendon tear and subscapularis tendinosis in one patient. Meanwhile, cases in the lower extremity group consisted of achilles tendon tear, ATFL tear, flexor digitorum longus tear, gastrocnemius partial tear, iliotibial band bursitis, labral tear hip, osteochondral defect, posterior tibialis tendon dysfunction, and lastly a case of both ATFL and CFL tear.

**Table I:** Subjects’ characteristics

	Baseline characteristics	N (%)
Sex	Male	34 (44.7)
	Female	42 (55.3)
Age (range: 15 - 83 years)	< 30 yrs.	10 (13.2)
	30-39 yrs.	11 (14.4)
	40-49 yrs.	11 (14.4)
	50-59 yrs.	23 (30.3)
	60-69 yrs.	16 (21.1)
	>70 yrs.	5 (6.6)
BMI	Underweight (< 18.5)	1 (1.3)
	Normal (18.5-22.9)	16 (21.1)
	Overweight (23-24.9)	21 (27.6)
	Obese I (25-29.9)	32 (42.1)
	Obese II (≥ 30)	6 (7.9)
Duration of Pain	Acute	31 (40.8)
	Chronic	45 (59.2)
Confounding Disease	Yes	43 (56.6)
	No	33 (43.4)
Site of Lesion	Upper Extremity	29 (38.2)
	Lower Extremity	47 (61.8)

**Fig. 1:**
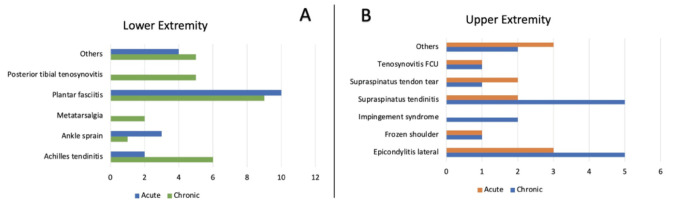
Distribution of soft tissue inflammation (upper extremity and lower extremity). (a) Chronic pain. (b) Acute pain.

The result of VAS evaluation is displayed in Table II. Patients in both acute and chronic group only reported that they felt mild to no pain after PRP injection (VAS: 0-3). Moreover, in the acute group there were only reports of pain with VAS scores of 0-2. The Paired Samples T-test revealed that both acute and chronic pain were significantly (p < 0.000) reduced after the injection (Table III).

**Table II:** VAS evaluation before and after PRP injection

	Before PRP Injection	After PRP Injection
**Acute Soft Tissue Inflammation**		
VAS (Mean ± SD)	3.06 (±1.28)	0.8 (±0.65)
Degree of Pain (N (%))	VAS	VAS
No pain	0 (0)	9 (29)
Mild pain (1-3)	21 (67.7)	22 (71)
Moderate pain (4-6)	10 (32.3)	0 (0)
Severe pain (>7)	0 (0)	0 (0)
**Chronic Soft Tissue Inflammation**		
VAS (Mean ± SD)	4 (±1.75)	0.97 (±0.62)
Degree of Pain (N (%))	VAS	VAS
No pain	0 (0)	9 (38.3)
Mild pain (1-3)	21 (46.7)	36 (61.7)
Moderate pain (4-6)	21 (46.7)	0 (0)
Severe pain (>7)	3 (6.6)	0 (0)

**Table III:** Paired T-Test evaluating the significance of PRP injection

		N	Mean VAS	Std Dev.	Mean difference	T	df	p value
Acute Pain	Before	31	3.06	1.28	2.25 (± 1.23)	10.16	30	<0.000
		After	31	0.80	0.65			
Chronic Pain	Before	45	4.00	1.75	3.02 (± 1.54)	13.125	44	<0.000
		After	45	0.97	0.62			

## Discussion

PRP’s efficacy comes from the various growth factors which are released from alpha granules of activated platelets, specifically platelet-derived growth factor (PDGF), epidermal growth factor (EGF), transforming growth factor-beta 1 (TGF-β1), vascular endothelial growth factor (VEGF), basic fibroblast growth factor (FGF), hepatocyte growth factor (HGF), and insulin-like growth factor (IGF-I)^2,3,6,7^. The degranulation also acts as chemoattractant for macrophages to take part in the healing process^8^. Based on previous literatures, increased platelet concentration is believed to escalate the total growth factors released, which enhance healing and the regeneration process^2,6,9,10^. The enhancement of the migratory ability leads to a better regeneration capability and slows down the natural progression of the disease. PRP works gently and will heal the microenvironment of the lesion^7^.

The characteristics of our subjects were similar to other patients in different centres^11^. We found that the majority of the patients with soft tissue injuries were female (42%). Although we did not account for any correlation between gender and PRP, previous studies reported that there was not enough evidence to suggest gender plays a significant role in the outcome of PRP therapy^12,13^. The patients’ age (40 - 69 years old) reveals that the lesions were mostly found in people who were still relatively active, which might impair their daily activities and will further worsen in time^13^. The predominant age may also reflect the cofounding diseases found in each patient. Diabetes and hypertension are notoriously frequent in this age range. Whilst this study did not correlate cofounding diseases with pain, the improvements in VAS score implied that systemic condition did not have an effect on PRP modulation. Inflamed tendon lesions are often instigated by chronic microinjuries due to excessive repetitive stress^10,14,15^. Patients whose injuries were sustained from jobs with extensive movement such as manual handling of heavy loads and demanding handgrip forces, or those with repetitive movement should be considered^11^ for this form of therapy. Certain activities or sports such as soccer and tennis are known to be the major causes of achilles tendinitis and lateral epicondylitis^14,16^. Some soft tissue injuries are also linked to high BMI. This may be caused by a higher loading force, particularly in the lower extremity of obese patients. Obesity is associated with prolonged low-grade inflammation and impaired insulin sensitivity^7^. In addition, enlargement of the diameter of tendon fibre with the shortening of its modulus could weaken the fibre’s ability to resist stress and reduce its healing process capacity. This is also supported by the fact that almost 70% of our patients were overweight and obese.

The pain relief may not be as potent as those from the use of corticosteroid or NSAID. However, PRP will stimulate the healing process^4^, thus the prolonged use of NSAID could be avoided. This was seen in various studies about plantar fasciitis which reported that PRP is capable of treating chronic pain^8,17,18^. After six months of follow-up, PRP showed a better outcome compared to corticosteroid injection in the management of elbow epicondylitis^19^. Conversely, there was no significant difference between PRP and corticosteroid in short to intermediate settings^17^. These findings were in concordance with our study where PRP has a significant value in treating both acute and chronic pain, with a considerably better result in chronic pain with a mean difference of 3.02 (±1.54).

Some surgeons are reluctant to prescribe PRP as a standalone treatment without additional analgesic regiment. This hesitation could be avoided by understanding the basic role and function of PRP. It is not necessary to inject the plasma directly to the tendon or ligament. Instead, the purpose of PRP injection is to enrich the surrounding inflamed locus. Our method is to inject it subcutaneously to the loose connective tissue, then gently massage the bump to disperse PRP evenly around the inflamed region. Another important element is to meticulously prepare the patient’s blood into a ready-to-use PRP that is then injected within five minutes before a clot could form.

This study had limitations as well. The design of this study is retrospective which could raise some biases and confounding factors which could influence the outcome. Further studies that can focus on one type of inflammation would be beneficial. Despite the favourable outcome, we acknowledge double-blinded RCTs with large sample sizes are needed before standardising PRP as the sole treatment modality for soft tissue inflammation.

## Conclusion

Our study indicates that PRP injection can provide both short-term and long-term relief in soft tissue musculoskeletal inflammation without any additional analgesic.
